# Melting and Rapid Solidification of Lunar Regolith Particles Returned by Chang’E-5 Mission

**DOI:** 10.34133/research.0486

**Published:** 2024-09-23

**Authors:** Xian Zhang, Yiwei Liu, Shaofan Zhao, Jian Song, Wei Yao, Weihua Wang, Zhigang Zou, Mengfei Yang

**Affiliations:** ^1^ Qian Xuesen Laboratory of Space Technology, China Academy of Space Technology (CAST), Beijing 100094, China.; ^2^ Institute of Physics, Chinese Academy of Sciences, Beijing 100190, China.; ^3^ College of Engineering and Applied Sciences, Nanjing University, Nanjing 210093, China.; ^4^ China Academy of Space Technology (CAST), Beijing 100094, China.

## Abstract

Melting and solidification of lunar regolith are pivotal for comprehending the evolutionary dynamics of lunar volcanism, geology, and impact history. Additionally, insights gained from these processes can contribute to the advancement of in situ resource utilization technologies, for instance additive manufacturing and resource extraction systems. Herein, we conduct the direct observation of the melting and rapid solidification of lunar particles returned by the Chang’E 5 mission. The melting temperature and melting sequence were obtained. Bubble generation, growth, and release were clearly observed, with a maximum bubble diameter of 5 µm, which is supposed to be according to the release of volatiles that embedded in the particles. During the solidification process, evident crystallization occurred with incremental crystal growth rate approximately of 27 nm/s. Scanning electron microscopy and energy-dispersive x-ray spectroscopy results verified that the Fe-rich mineral crystalizes first. These results would improve the understanding of the evolution of lunar volcanism, geology, and impact history.

## Introduction

Lunar regolith samples are of immense importance in providing key insights into the origins, geology, history of the Moon, as well as the evolution of the Solar System [1–3]. In the last century, Apollo and Luna missions have brought back ∼382-kg lunar regolith samples that have substantially enriched our knowledge of the lunar volcanism history [4,5], the mantle’s composition and structure [6–8], the space weathering of the lunar surface [9], and the influence of the lunar dust on lunar exploration [10,11]. Recently, the China’s Chang’E-5 mission successfully returned ~1.73-kg lunar regolith from the northeastern Oceanus Procellarum. Studying this new lunar regolith samples has led to a series of marked scientific findings [12–15], for instance, the unique physical properties at the new sampling site [16,17], the prolonged lunar volcanism with and non-KREEP origins [18], the glass fibers formed by relatively gentle impacts [19], the evidence of a previously unknown type of basaltic rock [20], the enriched He-3 in lunar regolith particles [21], and the origin of nano-Fe in glass beads [22]. Along with the scientific findings, lunar regolith samples also hold the potential to pave the way for future missions to the Moon, as well as the development of in situ resource utilization (ISRU).

Melting and solidification processes of lunar regolith play a critical role in comprehending the evolution of lunar volcanism, geology, and impact history. For instance, the agglutinates, which are bonded with smaller particles (mineral grains, glasses, and even older agglutinates) together by vesicular, flow-banded glasses, are formed by rapid melting and solidification of lunar regolith due to continuous micrometeoroid bombardment [23–25]. More importantly, understanding the properties and behavior of lunar materials during melting and solidification can also inform the development of ISRU technologies that rely on the properties of lunar materials, such as additive manufacturing, oxygen and volatile extraction, and metal extraction systems [26–30]. Substantial efforts have been dedicated to reproducing the dynamic crystallization of simulated lunar regolith samples [31–38]. These endeavors aimed to unravel the intricacies of basalt texture development, shedding light on what these textures can reveal about the formation processes of lunar lavas. These findings indicate that the formation of textures in basaltic rocks is controlled by factors such as nucleation and growth rates of crystals [35], cooling rates of lavas [39–43], composition of liquids [44,45], and the extent of fluid flow within the lavas [46–48]. However, these studies primarily concentrate on the crystallization at low cooling rates, according to the hypothetical cooling rate of the lunar mantle [45,49]. Unfortunately, slow heating and cooling rates may not accurately represent the properties and behavior of lunar regolith melts generated by small-to-moderate meteorite impacts [50]. These impact melts exhibit higher cooling rates compared to those of the lunar mantle, resulting in rapid solidification characteristics. Furthermore, in situ additive manufacturing (ISAM) technologies, such as selective solar light melting, also favor rapid melting and solidification processes. Therefore, there is an urgent need to explore the rapid melting and solidification of the lunar regolith samples. This exploration not only is crucial for understanding how small-to-moderate meteorite impacts contribute to the formation of agglutinates but also provides insights that guide the development of ISRU technologies.

Simulating lunar environments on Earth is challenging. Among the harsh conditions on the Moon, the vacuum plays a critical role in influencing the melting and solidification processes of lunar regolith. The absence of an atmosphere drastically changes heat transfer mechanisms, enhances the volatilization of certain elements, and influences the formation and behavior of gas bubbles within the melt. Additionally, it has been demonstrated that microgravity or low gravity does not evidently affect the melting and rapid solidification processes of lunar regolith simulant [51]. Herein, we conducted direct observation of the rapid melting and solidification processes under vacuum conditions using various lunar regolith particles returned by the Chang’E-5 mission. The melting sequence of various lunar particles has been determined. Distinctive processes, including unique crystallization, bubble generation, and explosive phenomena, were observed. These observations can help constrain the melt behaviors following small-to-moderate meteorite impacts, shedding light on the potential formation of vesicular agglutinates. This study holds important implications for comprehending the evolution of melts resulting from small-to-moderate meteorite impacts and providing valuable guidance for the advancement of ISRU technologies.

## Results

### Morphology and composition of different lunar regolith particles

The mineral analysis of the lunar regolith sample CE5C0400, including microscopy, scanning electron microscopy (SEM)-energy-dispersive x-ray spectroscopy (EDX), and powder x-ray diffraction, has been performed in our previous work [17]. According to this analysis, the vibrant particles identified in the lunar regolith sample CE5C0400 mainly consist of pyroxene (black or dark gray), plagioclase (off-white), olivine (yellowish green), ilmenite (black), glass fragments (brown), glass beads (yellow, brown, and black), and agglutinates (dark gray) [16,17]. Plagioclase and olivine particles are easily identified by their distinct colors, while glass beads (spherical and dumbbell-shaped) and the agglutinates (irregular shape) are easily chosen based on their characteristic shapes. The main difficulty lies in distinguishing between pyroxene and ilmenite. Fortunately, pyroxene–ilmenite intergrowths are often found in lunar basalts [38,52,53]. providing opportunities to select black particles containing both pyroxene and ilmenite.

**Fig. 1.**
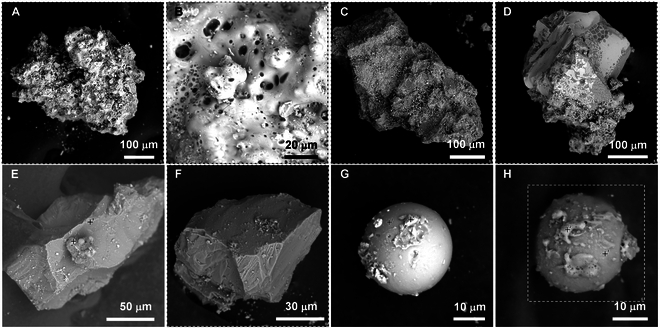
Morphology of the selected lunar regolith particles. SEM images of (A) an aggregate particle, (B) magnified area of the aggregate particle, (C and D) black basalt-based fragments, (E) a transparent plagioclase particle, (F) a yellowish-green olivine particle, (G) a yellow glass bead, and (H) a dark brown glass bead.

To further confirm the reliability of choosing particles based on their shapes and colors, we performed the SEM-EDX analysis. Figure 1 displays the SEM images of various lunar regolith particles selected from the lunar regolith sample CE5C0400 based on their shapes and colors. The agglutinate particle exhibits an irregular shape with an approximate size of 400 μm, as shown in Fig. 1A. Small grains are observed to adhere to its outer surface. The magnified image illustrates its porous structure, featuring hole diameters ranging from a few nanometers to several micrometers. Most of these holes have round or elliptical orifice with smooth edges. Therefore, it is reasonable to infer that the porous structure of the agglutinate is formed during the volatilization of low-boiling-point components. Elemental mappings and compositions of different lunar regolith particles obtained from the EDX results are summarized in Fig. S1 and Table. The agglutinate contains significant amounts of oxygen (O, ~65.9 at.%), silicon (Si, 13.4 at.%), aluminum (Al, 5.3 at.%), iron (Fe, 6.1 at.%), calcium (Ca, 3.0 at.%), magnesium (Mg, 4.4 at.%), and titanium (Ti, 1.4 at.%), with minor amounts of sodium (Na, 0.3 at.%) and potassium (K, 1.4 at.%). Notably, bright Fe and Ti spots were observed, which correspond to adhering ilmenite grains.

**Table.** Elemental contents of the selected Chang’E-5 lunar regolith particles acquired from EDS mapping

Elements	Atomic ratio (%)						
	Particle 1: agglutinate	Particle 2: basalt fragment	Particle 3: basalt fragment	Particle 4: plagioclase	Particle 5: olivine	Particle 6: glass bead (yellow)	Particle 7: glass bead (brown)
O	~65.9	~67.5	72.7	~69.3	~60.9	~69.5	~67.1
Si	13.4	14.0	9.2	13.7	12.1	10.0	12.9
Al	5.3	7.0	2.9	9.7	1.5	5.1	5.5
Ca	3.0	3.7	1.4	3.9	0.6	3.1	3.2
Fe	6.1	4.4	9.7	1.4	11.3	7.7	5.8
Mg	4.4	2.2	1.1	0.5	13.6	3.5	3.3
Ti	1.4	0.5	3.0	0.2	-	1.1	1.4
Na	0.4	0.5	-	1.2	-	-	1.0
K	0.1	0.2	-	0.1	-	-	-

Figure 1C and D display 2 black basalt-based fragments. Similar to the agglutinate particle, these 2 fragments also exhibit irregular shapes with sizes ranging from 300 to 500 μm. However, they possess compact structure with evident rock texture and crystal grains. The fragment in Fig. 1D also has some flow-banded glass on its surface, suggesting signs of surface melting. The composition of the fragment in Fig. 1C is similar to the average composition of lunar regolith, as summarized in Table, indicating that it is predominantly composed of pyroxene. The gathering of Al, Fe, and Mg in the elemental mapping of the fragment in Fig. 1C suggests the presence of plagioclase and olivine grains. Elemental distribution of the fragment in Fig. 1D shows the evident pyroxene–ilmenite intergrowth phenomenon, as illustrated in Fig. S1C. Besides, the olivine grain is iron-enriched with a Fe/Mg ratio of 20.2/3.6, indicating that this fragment crystallized at a low cooling rate [54]. In addition, K-rich regions, i.e., potassium feldspar, were also observed in this fragment, appearing as small interstitial lath crystals (Fig. S2). These phenomena also confirmed that the fragment formed in the middle to late period of lava cooling [18,45,54].

Figure 1E and F show a transparent plagioclase particle and an olivine particle, respectively. These 2 particles are typical crystal fragments with clean cleavage planes. The major elements distribute uniformly in the transparent particle (Fig. 1E and Fig. S1D) and the atomic ratio of (Ca, Na)/(Al,Si) is 5.1/23.4, close to 1/4, further confirming that the particle is typical plagioclase ((Ca,Na)(Al,Si)_4_O_8_). Likewise, the atomic ratio of (Mg, Fe)/Si in the particle in Fig. 1F is 24.9/12.1, close to 2/1, further confirming that this particle is typical olivine ((Mg,Fe)_2_SiO_4_, Fa_32_).

In addition to the pyroxene, plagioclase, ilmenite, olivine, and agglutinate, the lunar regolith particles also contain many glass beads, as shown in Fig. 1G and Fig. 1F. These 2 beads are both about 30 μm in diameter. In comparison, the glass bead in Fig. 1G exhibits a much more spheroidal shape with a cleaned and smooth surface. The glass bead in Fig. 1H is surrounded by numerous solidified lava formations. The primary composition of the 2 glass beads is comparable, although the glass bead in Fig. 1G contains a significantly higher iron content than another. Based on the previous work [17] and the SEM-EDX results, we can select individual particles based on the shapes and colors for the subsequent destructive tests.

### Melting and solidification process of lunar regolith particles

#### Pyroxene and ilmenite

**Fig. 2.**
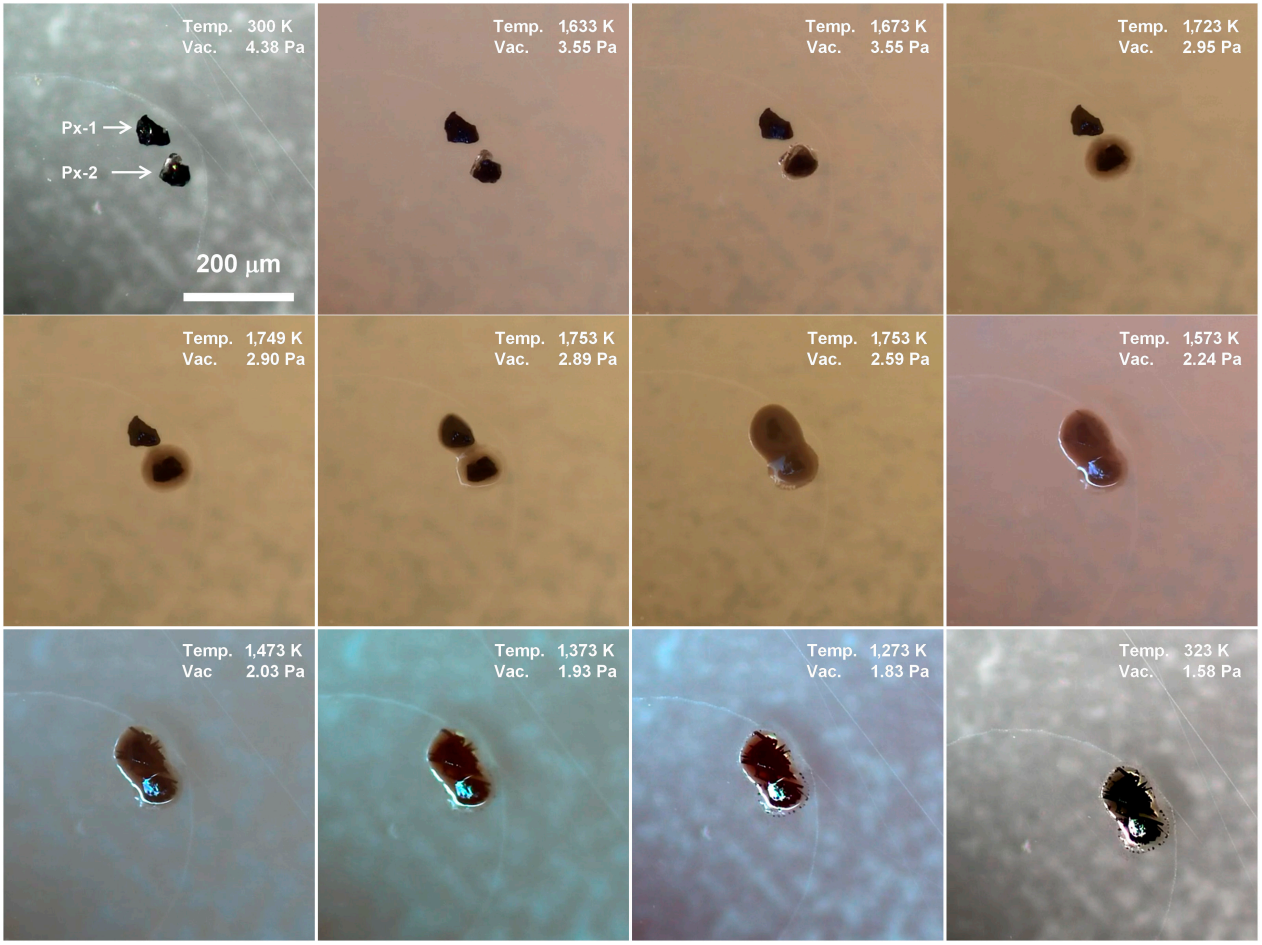
Micrographs of 2 pyroxene particles (Px-1 and Px-2) at different temperatures.

As the most prevalent mineral in lunar regolith, gaining insight into the melting and solidification behavior of pyroxene is of great importance [16,17]. Figure 2 shows the micrographs of 2 pyroxene particles (Px-1 and Px-2) captured at different temperature. The 2 pyroxene particles are about 60 μm in size. During the heating process, the Px-2 initially showed noticeable rounding as the temperature reached 1,633 K and then partially turned to liquid state at 1,673 K, accompanied by a black dystectic grain. Further temperature increase resulted in the surface melting of the Px-1. The liquid gradually spread to the sapphire substrate due to the decrease of viscosity, followed by merging with Px-2 at 1,753 K. The 2 black dystectic grains then dissolved slowly at 1,753 K, and the melt turned to dark brown. The main dark minerals in lunar regolith include pyroxene and ilmenite, with ilmenite having a higher melting point than pyroxene. Therefore, it is reasonable to infer that the black dystectic grains are ilmenite. The 2 pyroxene particles did not form a homogenous melt after been maintained at 1,753 K for 4 min. During the cooling process, multiple needle-like black crystals emerged in the melt at around 1,573 K. These crystals then experienced rapid growth from 1,573 to 1473 K. In the final stage of solidification, some black dots appeared at the edge of the melt.

**Fig. 3.**
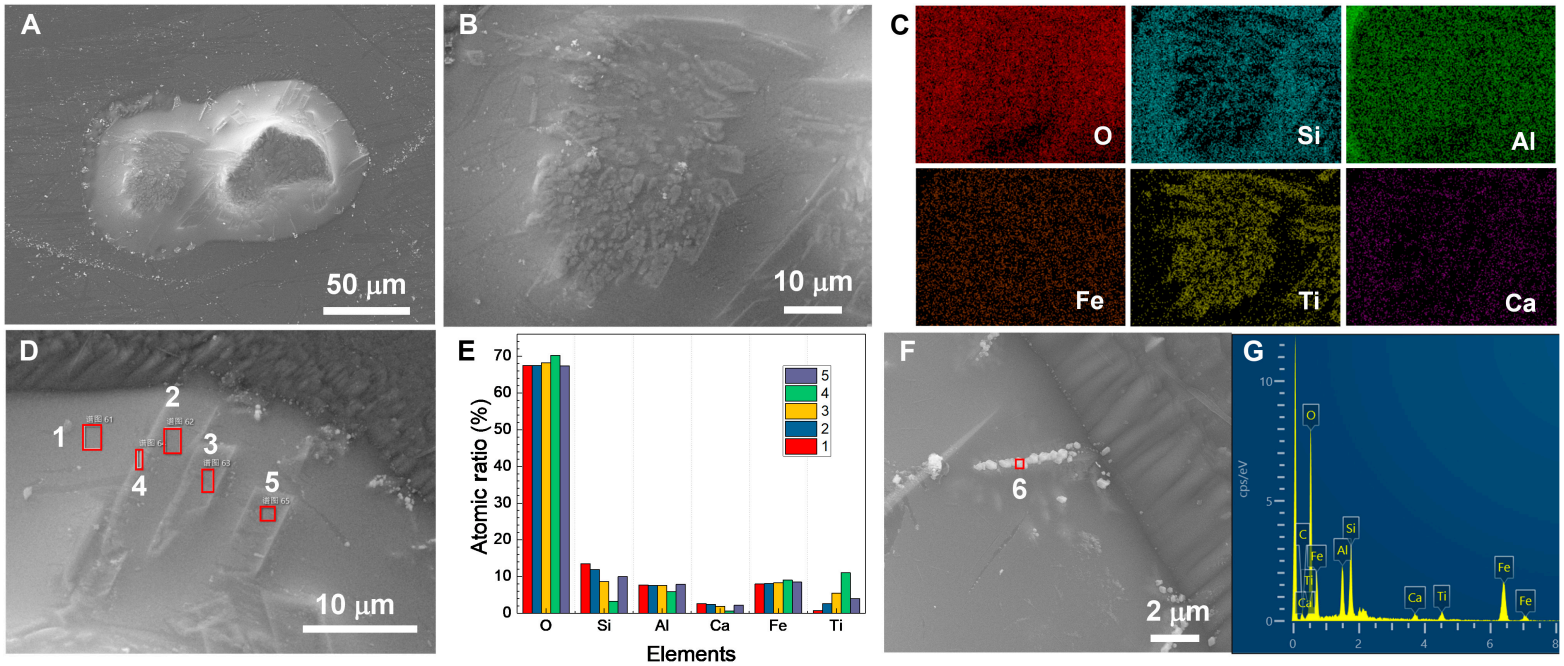
Morphology and composition of the solidified pyroxene melt. (A) SEM image of the molten pyroxene particles after cooling. (B and C) The SEM image and elemental distribution of the top of the solidified melt. (D) SEM image of the crystals at the edge of the solidified melt. (E) Atomic ratio of the 1 to 5 regions. (F) Bright grains on the upper surface at the edge of the melt. (G) EDX spectrum of the region 6.

Figure 3 illustrates the morphology and EDX results of the solidified melt of the 2 pyroxene particles. In the low-magnification image (Fig. 3A), crystals grown from the melt and undissolved eutectic grains are easily discernible. Notably, the sizes of the 2 black dystectic grains after complete solidification are larger than that at 1,753 K, indicating rapid crystal growth during the cooling stage. In addition, the newly grown crystals are tightly surrounded by solidified melt, forming a glass–ceramic-like structure (Fig. 3B). The elemental distribution of the region confirms a pronounced accumulation of Ti, while Fe is uniformly distributed, as shown in Fig. 3C. This phenomenon suggests that Fe has higher solubility in the melt compared to Ti. At the edge of the melts, needle-like crystals were also observed (Fig. 3D), which are also enriched in Fe and Ti (Fig. 3E). Hence, it can be concluded that the black crystals growing from the melt are ilmenite. Moreover, some bright grains with a lined arrangement appeared on the upper surface at the edge of the melt, as illustrated in Fig. 3F. These small crystals are Fe-enriched grains (Fig. 3G). At the interface between the melt and the substrate, there are also some Al_2_O_3_ whiskers (Fig. S3), attributed to the erosion of the melt at high temperature.

#### Plagioclase

**Fig. 4.**
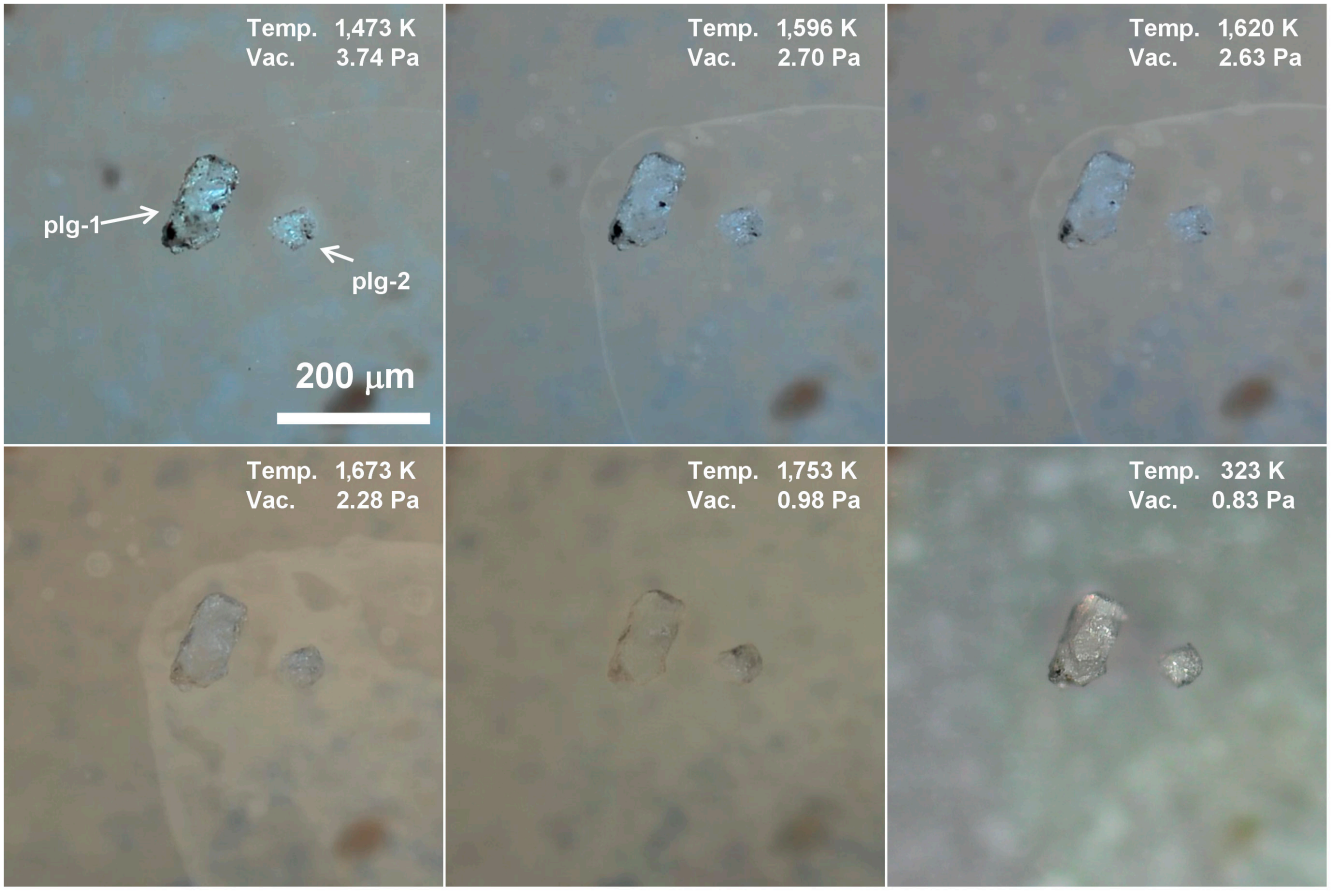
Micrographs of 2 plagioclase particles (plg-1 and plg-2) at different temperature.

The second most abundant mineral in lunar regolith is plagioclase, which contains about 40% of Al_2_O_3_ of the whole Moon [54]. Figure 4 illustrates the micrographs of 2 transparent plagioclase particles (plg-1 and plg-2) at various temperatures. The plg-1 is a lath crystal with black particles adhering to its surface, while the plg-2 is a white aggregate of plagioclase grains. These 2 plagioclase particles remain unchanged in the temperature range of 300 to 1,473 K, illustrating their excellent thermal stability. Further elevating the temperature results in a reduction in the transparency of plg-1. When the temperature reaches 1,596 K, the black particles on plg-1 begin to melt, and it transitions into a liquid state, wetting the lath crystal at 1,673 K. However, there is no evident sign that either of the 2 plagioclase particles would undergo melting, even when the temperature reaches 1,753 K. This phenomenon is attributed to the fact that the plagioclase particles are mainly composed of high-melting lithophile (Ca, Si, Al, etc.). During the cooling stage, the melt of the black particles solidified, forming a new black particle on plg-1. It is worth noting that both plagioclase particles exhibit slightly glossier surface after cooling, indicating inconspicuous surface melting.

**Fig. 5.**
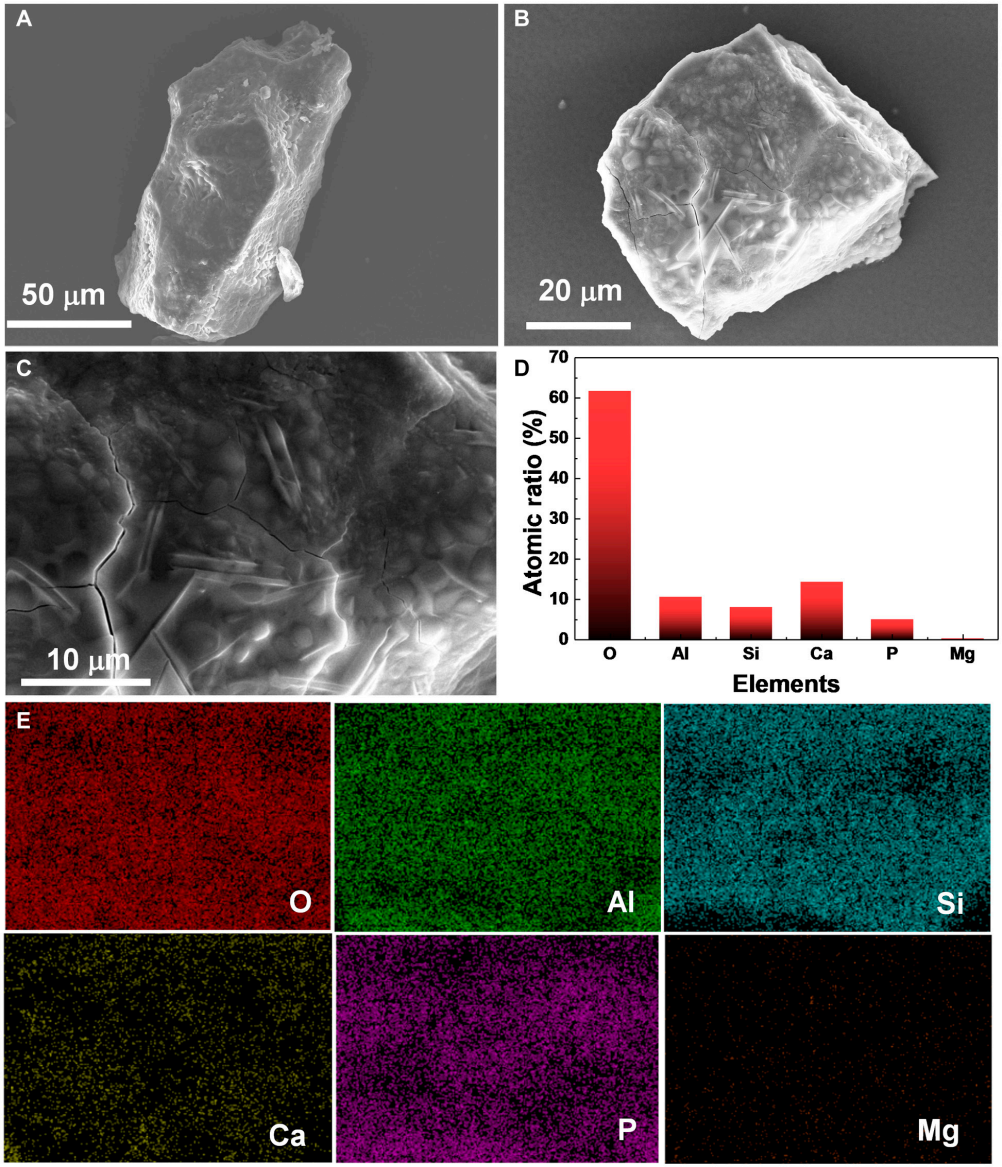
Morphology and composition of the plagioclase particles. (A and B) SEM images of 2 plagioclase particles after heat treatment. (C) Magnified area of the plg-2 surface. (D and E) Atomic ratio and elemental mapping of the magnified area of the plg-2 surface.

Figure 5 shows the SEM and EDX results of the 2 plagioclase particles after heat treatment. Both particles exhibit a rough surface with differently shaped small grains embedded in solidified melt, resulting from the surface melting (Fig. 5A and B). EDX results reveal that the particles are abundant in Ca and Al but have very low Fe and Ti content (Fig. 5D). Additionally, the elements are uniformly distributed, further confirming that the 2 particles are typical plagioclase. The composition of the particle surface shows an unexpected high atomic ratio of P, possibly due to the presence of unknown phosphorylated impurities from the substrate (Fig. S4). Moreover, evident cracks were observed in both particles, attributed to the thermal stress during heating and cooling.

#### Glass

**Fig. 6.**
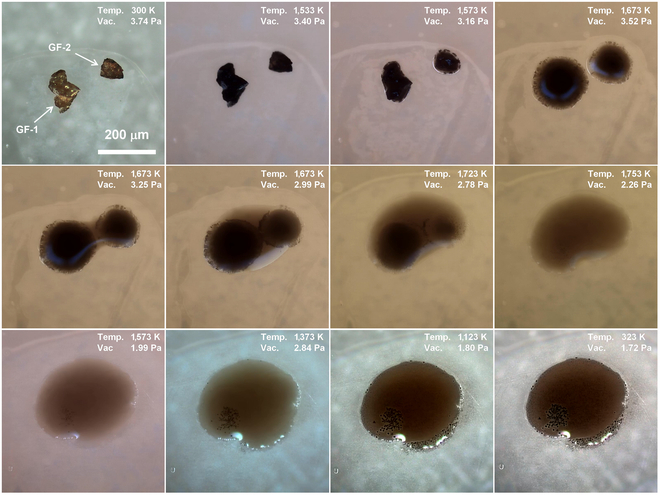
Micrograph of 2 glass fragments (GF-1 and GF-2) during heating and cooling process.

Our previous work has confirmed that the lunar regolith contains about 15% of glass [17]. Hence, it is necessary to observe the melting and solidification behavior of glasses. Figure 6 shows the melting and solidification process of 2 brown glass fragments (GF-1 and GF-2). Before heating, the 2 glass fragments have a dark brown color. Subsequently, the transmittance of the glass fragments was dramatically reduced during the heating process. The fragments began to shrink around 1,533 K and showed obvious melting at 1,573 K. Then, the 2 fragments turned to liquid state at 1,673 K with black dystectic grains embedded in the molten state. The formation of black dystectic grains is the main reason for the reduction in transmittance. Maintaining the temperature at 1,673 K, the liquid slowly spread to the substrate and merged with each other. The black dystectic grains did not transfer with the liquid, leaving 2 black circles under the molten state. With a further increase in temperature, the black dystectic grains dissolved slowly, forming a roughly homogenous liquid at 1,753 K. During the cooling process, the black grains recrystallized at 1,573 K. After complete solidification, a new dark brown glass with black crystals sinking to the bottom has formed.

**Fig. 7.**
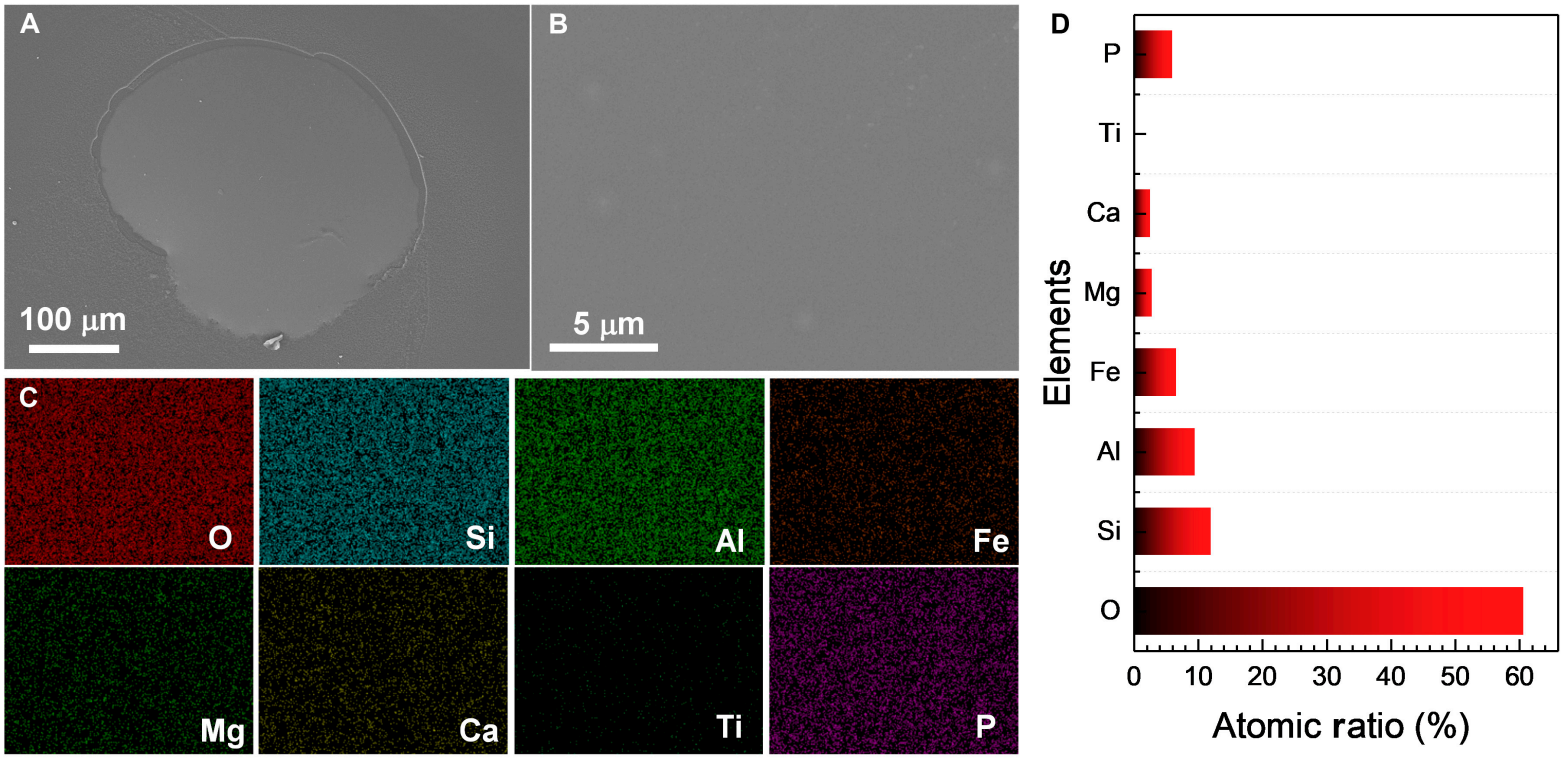
Morphology and composition of solidified glass melt. SEM images (A and B), elemental mapping (C), and the atomic ratio (D) of the melt of 2 glass fragments after cooling.

SEM images of the solidified melt with a smooth surface further confirm its glassy nature, as shown in Fig. 7A and B. The major elements are uniformly distributed in the melt (Fig. 7C). The 2 glass fragments revealed a low Ti content (0.14 at.%), as shown in Fig. 7D, compared to the composition of lunar regolith sample CE5C0400 [17]. The black grains were not visible in the SEM image, suggesting that they sank to the bottom of the liquid. Some highlighted circles were observed on the upper surface, but the elemental mapping did not show any marked accumulation (Fig. 7C). Overall, the dark brown glass fragments formed a roughly homogenous glass after melting and solidification.

**Fig. 8.**
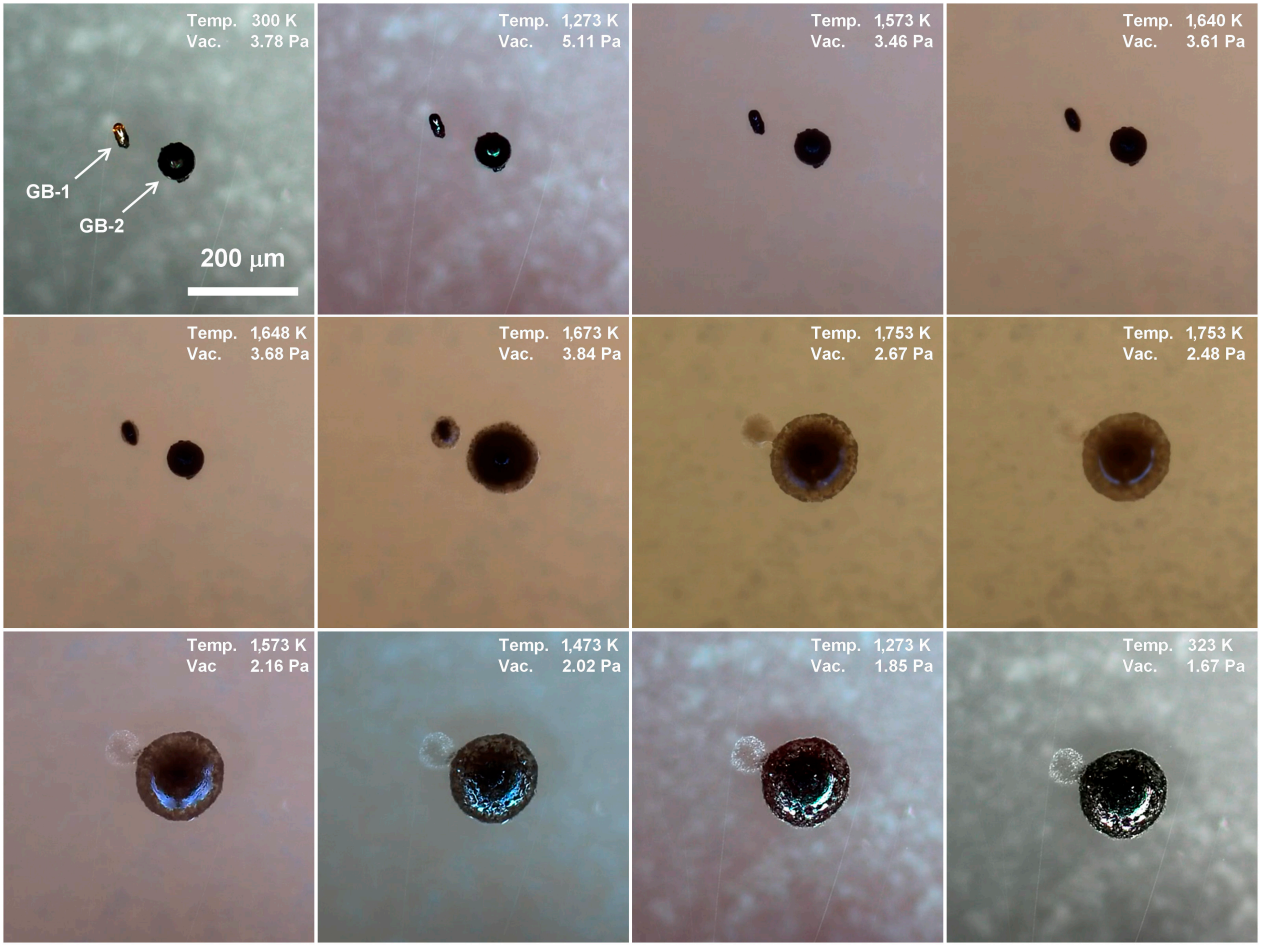
Micrograph of 2 glass beads (GB-1 and GB-2) during heating and cooling processes.

In addition to the dark brown glass fragments, there are glass beads in the lunar regolith that cannot be ignored. Figure 8 shows micrographs of 2 selected glass beads (GB-1 and GB-2) during heating and cooling processes. The glass beads have smooth surfaces but are typically adhered by solidified lavas. The GB-1, with a cylindrical shape, began to round at 1,573 K, and noticeable melting behavior was observed at 1,640 K. Subsequently, the solidified lavas on GB-2 shrank at 1,648 K, followed by the melting of the GB-2 at 1,673 K. Black dystectic grains, similar to those observed in the molten glass fragments, were also present in the melt of GB-2. The melt of GB-1 became transparent at 1,753 K. However, the melt of GB-2 remained opaque, which may be due to the high content of Fe and Ti. In addition, the melt of GB-1 was quickly absorbed by the melt of GB-2 at 1,753 K. The melt became coarse between 1,573 and 1,473 K, suggesting surface crystallization. After solidification, the GB-2 formed a black melt with a coarse surface, while only a few white crystals remained at the location of the GB-1.

**Fig. 9.**
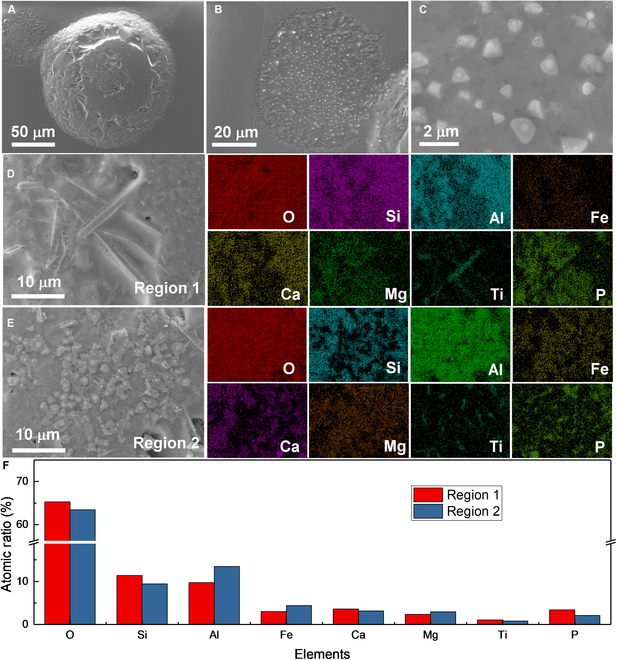
Morphology and composition of the solidified melt of glass beads. (A) SEM image of the melt formed by GB-2 after cooling. (B) SEM image of the melt formed by GB-1 after cooling. (C) Triangular plate crystals at the GB-1 site. (D) SEM and elemental mapping of the region 1 with needle-like crystals. (E) SEM and elemental mapping of the region 1 with triangular plate crystals. (F) Atomic ratio of region 1 and region 2.

Figure 9A to C display the SEM images of the products obtained from the melting and solidification of the 2 glass beads. Notably, a partially amorphous phase remained at the top of the melt. The melt exhibits erosional effects on the substrate at high temperature, as clearly shown in Fig. 9B. Two primary types of crystals are observed in the melt: needle-like crystals (Fig. 9D) and triangular plate crystals (Fig. 9E). These crystals are tightly bonded by solidified melt, forming a glass–ceramic-like microstructure similar to that of pyroxene. Elemental mapping results reveal that the needle-like crystals are rich in Ti, while the triangular plate crystals are rich in Al, Fe, and Mg (Fig. 9D to F). Additionally, some plagioclase lath crystals embedded in the melt were observed. Interestingly, many of the triangular plate crystals are adhered by spherical particles, which are the residual melt (Fig. S5).

Upon comparing the atomic ratios between melts of the glass beads and the glass fragments, the primary difference is the Ti content, as shown in Fig. S6. The Ti content in the melt of the glass beads is approximately 6 times higher than that in the melt of the glass fragments. This elevated Ti content promotes the crystallization of Ti-bearing minerals in the glass bead melts, resulting in a black appearance. In contrast, the crystallization in the melt of the glass fragments is evidently reduced despite their high Fe content.

#### Agglutinates and mixture

**Fig. 10.**
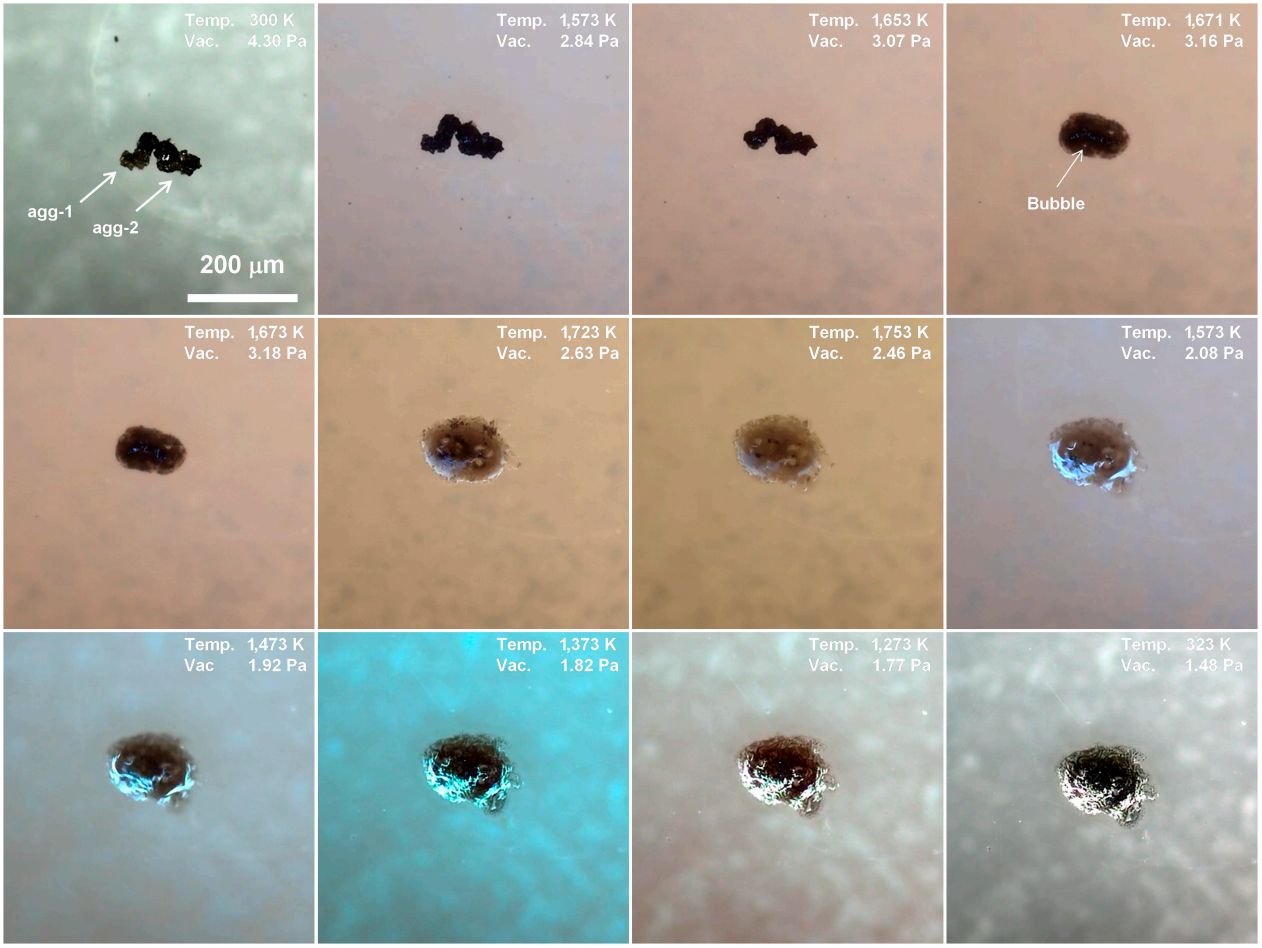
Micrograph of 2 agglutinates (agg-1 and agg-2) during heating and cooling processes.

As discussed above, the agglutinates are formed by bonding smaller particles (mineral grains, glasses, and even older agglutinates) together with vesicular, flow-banded glasses. Therefore, the melting and solidification behavior of agglutinates can provide insights into the behavior of lunar regolith. Figure 10 illustrates the melting and solidification processes of 2 typical agglutinates (agg-1 and agg-2). They both have irregular shapes with sizes of about 80 μm. During heating, the agglutinates began to shrink around 1,500 K, with a size reduction of approximately 50% at 1,653 K. The 2 agglutinates then turned into liquid and rapidly spread across the substrate. A bubble was observed in the melt at 1,668 K and then released at 1,670 K. As the temperature increased to 1,753 K, the black dystectic grains gradually dissolved, and several transparent regions appeared in the melt. These transparent regions are plagioclase particles that are wrapped in the 2 agglutinates. After holding at 1,753 K for 4 min, most of the black dystectnic grains dissolved, improving the transmittance of the melt. However, the melt did not become a homogenous liquid. During the cooling stage, the black grains began to recrystallize at 1,573 K. In addition, surface reflections became more intensive upon cooling. This phenomenon is attributed to the crystallization in the melt, leading to the formation of numerous crystal–liquid interfaces that increased light reflection. The black crystals in the melt grew rapidly between 1,573 and 1,373 K, forming a glass–ceramic-like microstructure. Afterwards, the melt remained essentially unchanged before it completely solidified.

**Fig. 11.**
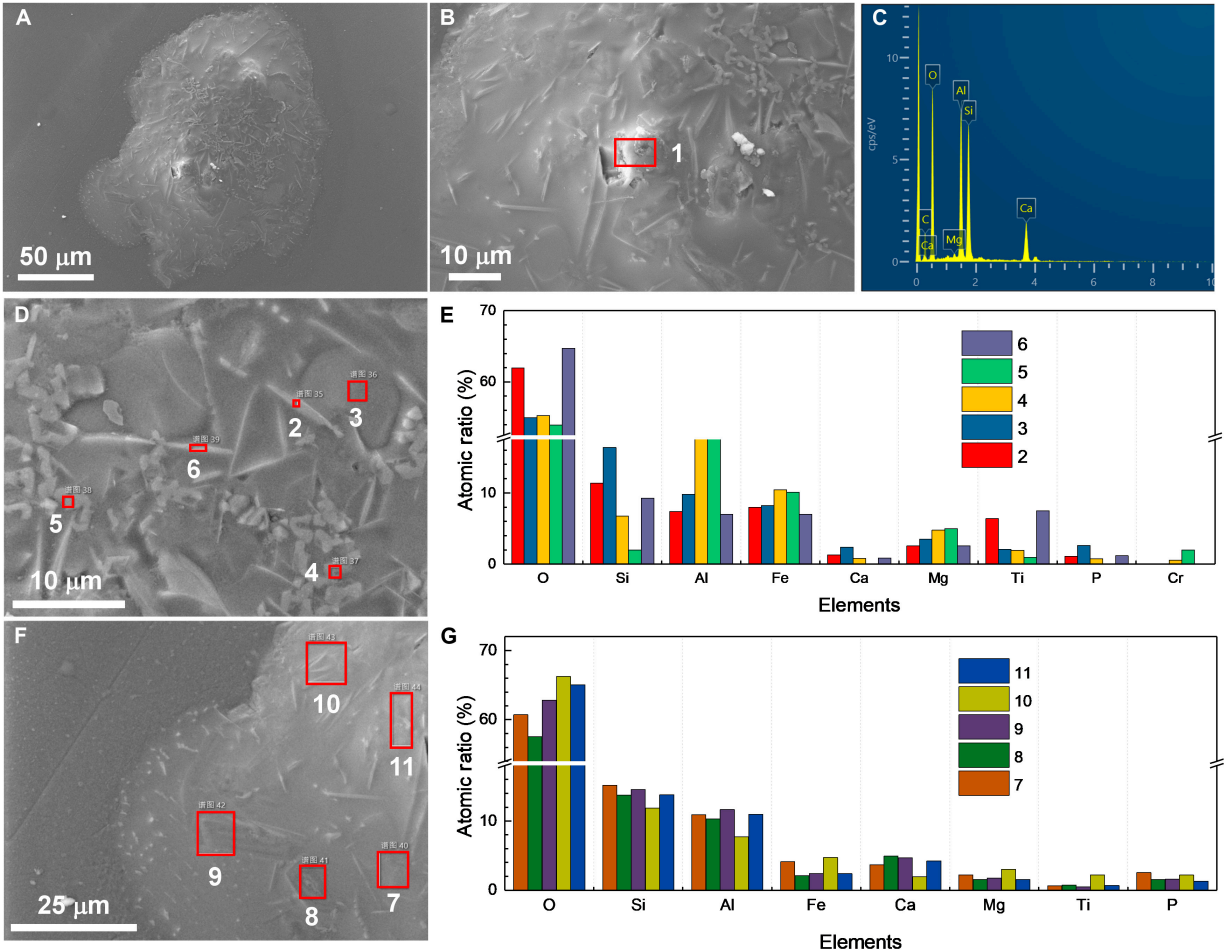
Morphology and composition of solidified melt of agglutinates. (A) SEM image of the melt of 2 agglutinates after cooling. (B and C) SEM image and EDX spectrum of the protruding region. (D and E) SEM image and the atomic ratio of the region abundant with needle-like crystals. (F and G) SEM image and the atomic ratio of the region abundant with lath crystals.

SEM images and EDX results of the solidified melt obtained from heating the 2 agglutinates are shown in Fig. 11. The low-magnification image reveals a variety of microstructures, including needle-like crystals, lath crystals, small grains, and solidified melt, as depicted in Fig. 11A. Several protruding regions observed in the solid are attributed to the unmolten plagioclase particles that emerged in the melt at 1,723 K. The major elements in these regions (Ca, Si, Al, and O) confirm their origin as residues of plagioclase particles, as shown in Fig. 11B and C. The needle-like crystals are rich in Fe and Ti, while the small grains are predominantly composed of Mg, Fe, Al, and O, as illustrated in Fig. 11D and E. These phenomena are consistent with observations made in the melt of glass beads. The interstitial regions between the needle-like crystals are mainly composed of Si and Al. Additionally, lath crystals, found near the protruding regions (Fig. 11F), are composed mainly of Ca, Al, Si, and O (Fig. 11G), indicating they are recrystallized plagioclases. Overall, the obtained solidified melt consists of plagioclases, ilmenites, and spinels embedded in a molten matrix, resulting in a glass–ceramic-like microstructure.

**Fig. 12.**
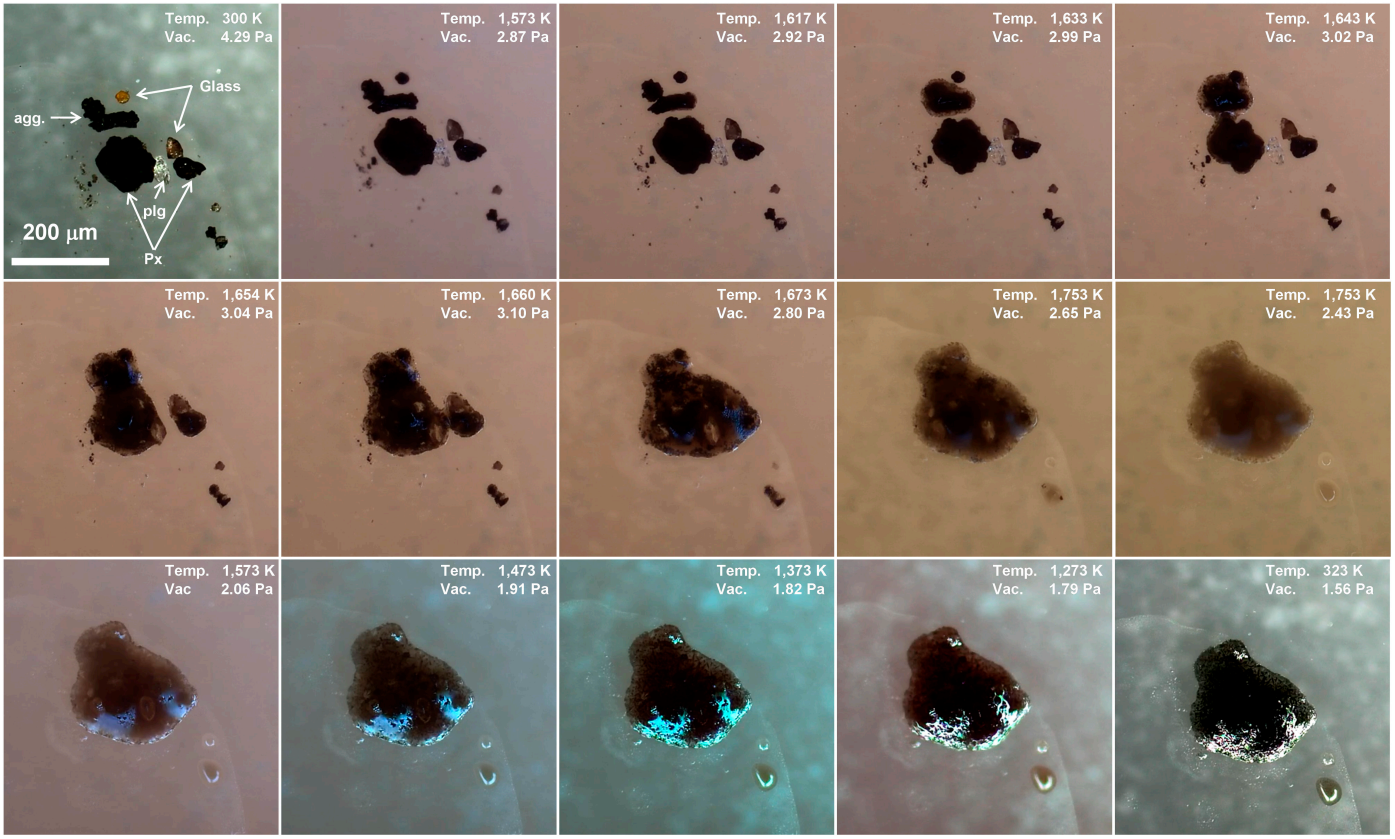
Micrograph of several selected lunar regolith particles, including 2 pyroxene particles (Px), a plagioclase particle (plg), an agglutinate particle (agg.), 2 glass fragment, and several small grains, during heating and cooling processes.

To gain further insight into the melting and solidification mechanisms of lunar regolith, we conducted heat treatment on a selection of various lunar regolith particles, as illustrated in Fig. 12. The mixture includes 2 pyroxene particles, a plagioclase particle, an agglutinate particle, 2 glass fragments, and several small grains that could not be identified under the microscope. The particle combination was chosen according to the compositions of the lunar regolith sample CE5C0400. The agglutinate particle began melting at 1,617 K and then transitioned to a liquid state at 1,633 K. The resulting melt continuously spread onto the substrate and absorbed the orange glass fragment at 1,643 K. During these processes, the pyroxene particles started melting and merged rapidly with adjacent particles/melts as well as with each other. Notably, a bubble emerged and expanded in the temperature range from 1,654 to 1,660 K, releasing before reaching 1,673 K. When the temperature reached 1,753 K, the melt did not homogenize into a universal liquid, as the plagioclases and black grains were still distinctly observed within it. However, maintaining this temperature caused the residual plagioclases and black grains to gradually dissolve, resulting in an improvement in the transparency of the melt. During the cooling stage, the melt exhibited similar solidification behaviors to those of the agglutinates. Needle-like crystals grew rapidly in the temperature range of 1,573 to 1,373 K. Abnormal surface reflections also suggested surface crystallization. After complete solidification, a solid with a glass–ceramic-like microstructure was obtained. In addition to this solid, 2 transparent drops were also formed.

**Fig. 13.**
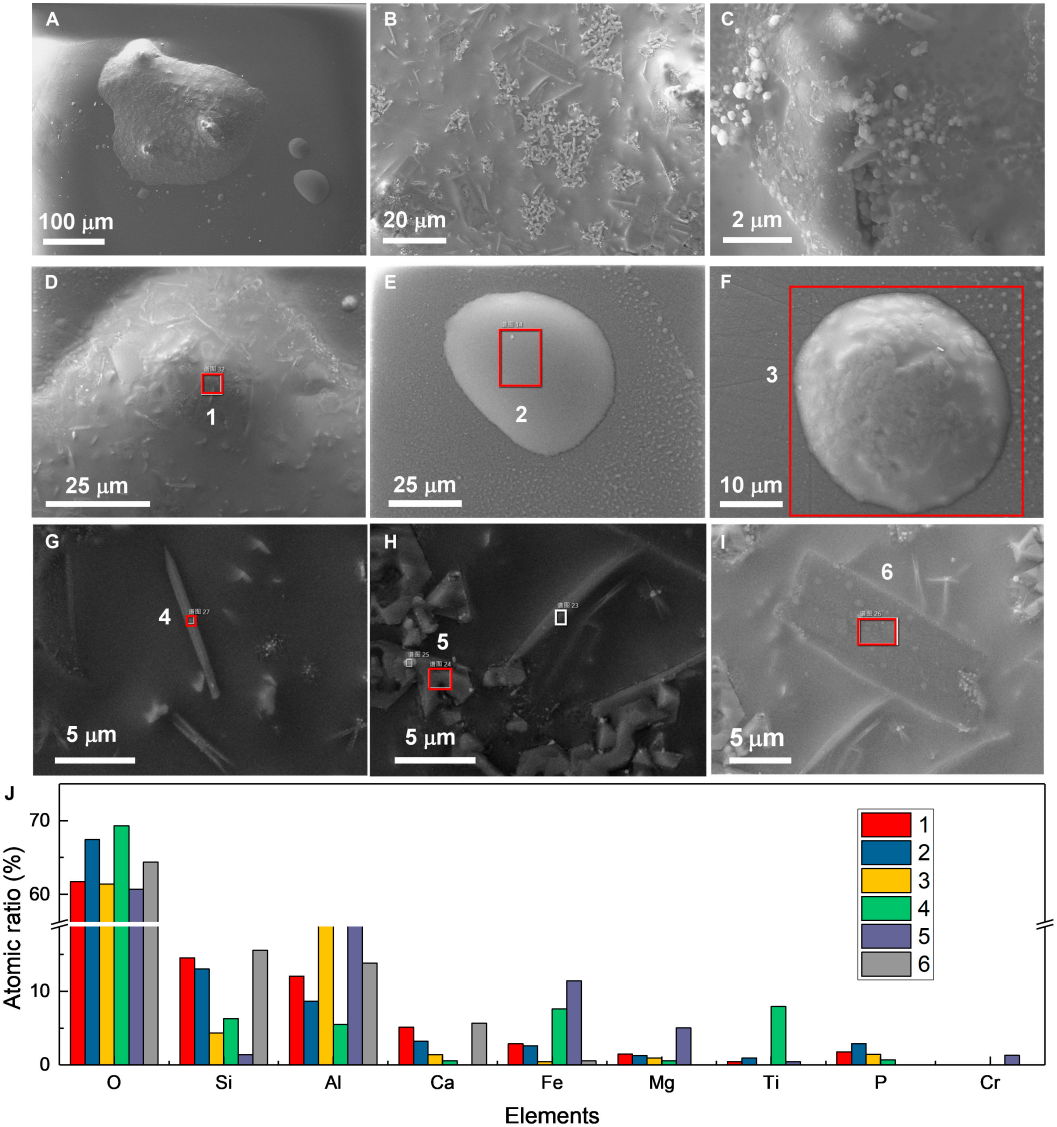
Morphology and composition of the solidified melt of the mixture. (A to C) SEM images of the melt of several different lunar regolith particles after cooling. (D) SEM image of a protruding region. (E and F) SEM image of the 2 droplets. (G to I) SEM image of the needle-like crystal, small grains, and lath crystals. (J) Atomic ratio of the regions marked by red rectangles.

SEM-EDX analysis were performed to characterize the morphology and composition of the formed solid, as shown in Fig. 13. The low-magnification image reveals a large solidified melt and 2 small droplets. Intriguingly, 3 protruding regions appears in the solid. The first is located at the top of the image and corresponds to the gathering of plagioclases. EDX results reveal that the primary components of this region are Ca, Al, Si, and O, as shown in Fig. 13D and J. The other 2 protruding regions are attributed to the residual plagioclase particles that were not completely dissolved in the melt. Similar to that of the agglutinates, numerous needle-like crystals, small grains, and lath crystals are present in the solidified melt. The 2 droplets exhibit different morphologies: one has a smooth surface while the other displays a coarse surface. The large droplet is completely glass with elements distributed uniformly (Fig. 13E and J). Interestingly, both droplets lack Fe and Ti. The composition of the needle-like crystals, small grains, and lath crystals was also examined. Similar to that of the agglutinates, the needle-like crystals are rich in Fe and Ti (Fig. 13G and J), while the small grains are mainly composed of Mg, Fe, Al, and O (Fig. 13H and J). The lath crystals are identified as recrystallized plagioclases (Fig. 13I and J).

## Discussion

### Crystallization

Crystallization of the lunar melts is crucial to understanding how the textures in basalts develop and what they can reveal about how the lavas formed. The typical crystallization sequence of lunar basaltic melts is as follows: olivine forms first, followed by pyroxene, plagioclase, and finally ilmenite [45]. This sequence results from the gradual cooling of the basaltic melt, which allows the different minerals to crystallize in a particular order based on their melting temperatures and other physical properties. However, according to the composition analysis of the products obtained from the melting and rapid solidification processes, the crystals were predominantly ilmenite instead of olivine. This abnormal crystallization pattern may be due to the rapid cooling and high concentration of titanium (Ti) in the matrix, which can accelerate the crystallization process of Ti-bearing minerals. Besides, the relative low Mg index also contributed to the deviation from the expected crystallization sequence.

**Fig. 14.**
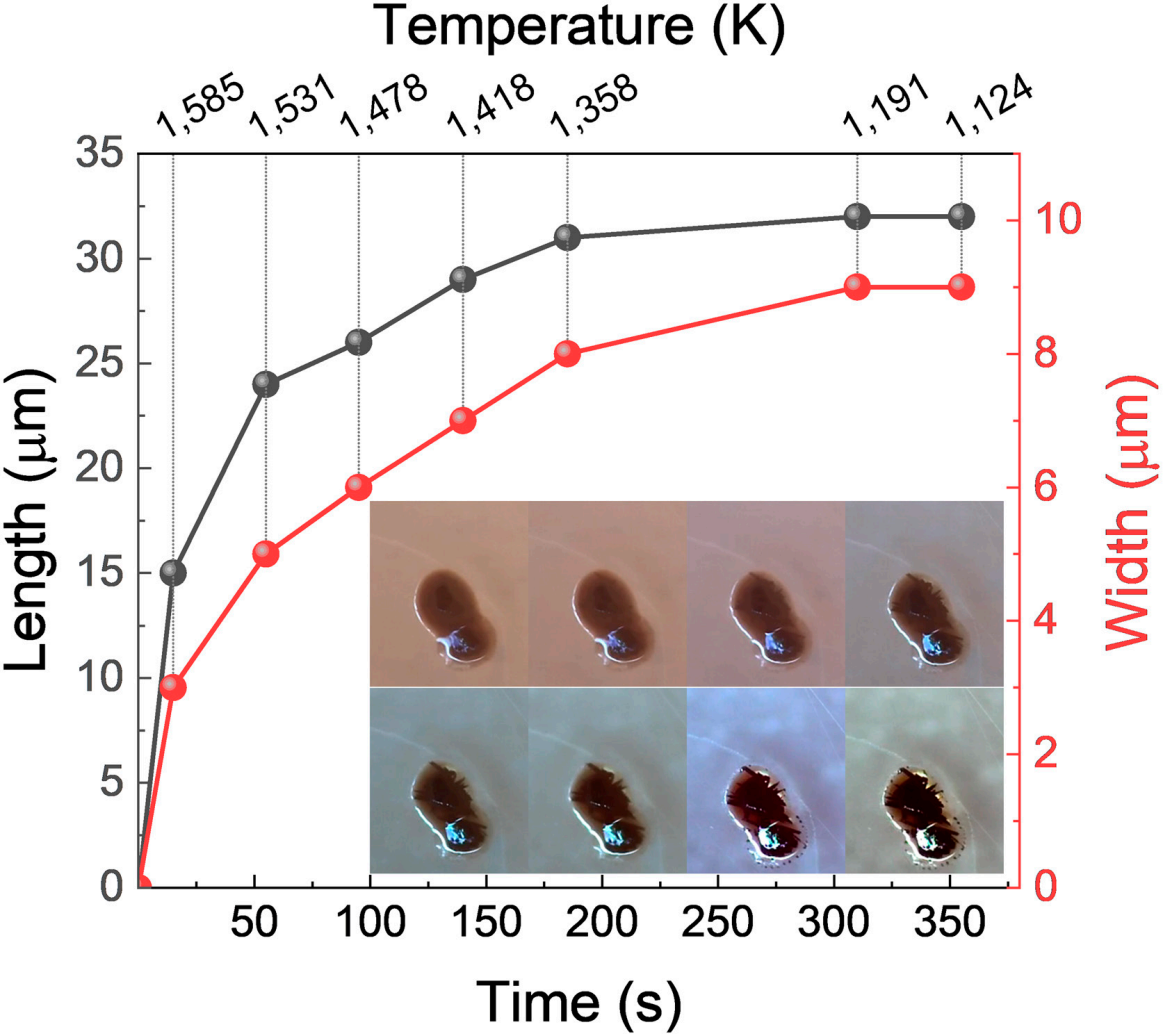
Crystal growth in the melt of pyroxene and ilmenite upon cooling.

Figure 14 shows the growth curve of a typical crystal in the melt of pyroxene and ilmenite upon cooling. The first signs of crystallization appeared at 1,611 K during cooling (defined as *t* = 0). Subsequently, only the growth of already existing crystals was observed, with no new crystals forming. The crystal grew rapidly after nucleation (1,611 to 1,531 K) and then entered a relatively steady growth period. Eventually, the crystal stopped growing, not only due to the rapidly increased viscosity but also due to the volume limitation of the matrix. The maximum growth rate was calculated using the following relationships:

$$Y_{L}=0.5L/t$$ (1)

where *L* is the crystal length, and *t* is the duration of the experiment [55,56]. The incremental growth rate *Y_Li_* was calculated from the relationship:

$$Y_{\mathrm{Li}}=0.5\left(L_{2}-L_{1}\right)/\left(t_{2}-t_{1}\right)$$ (2)

where *L*_2_ and *L*_1_ are the maximum lengths measured at experimental times *t*_2_ and *t*_1_, respectively [55,56]. The maximum growth rate of the chosen crystal is about 84 nm/s, while the *Y_Li_* in the temperature range of 1,531 to 1,358 K is about 27 nm/s.

Crystal growth kinetics of silicate melts has been investigated by many groups. Le Gall et al. [57] have studied the in situ crystallization kinetics of plagioclase and clinopyroxene in basaltic melt and derived a crystal growth rate of ~80 nm/s based on Eq. 1. Giuliani et al. [58] have studied the chemical variations of spinel, clinopyroxene, and plagioclase in MORB basaltic melt induced by continuous cooling and observed a maximum crystal growth rate of 25 nm/s for clinopyroxene at a cooling rate of 3 K/min. Iezzi et al. [59] have studied the solidification of an andesitic melt by cooling and observed the maximum crystal growth rate of 24 nm/s at a cooling rate of 25 K/min. Pontesilli et al. [60] have studied the crystallization kinetics of clinopyroxene and titanomagnetite growing from a trachybasaltic melt, observing maximum crystal growth rate of 18 nm/s for clinopyroxene and 2 nm/s for titanomagnetite. Arzilli et al. [61] have studied the crystallization kinetics of alkali feldspars in trachytic melt, observing a maximum crystal growth rate of ~10 nm/s and incremental growth rate varying from 0.1 to 10 nm/s. They also observed the incremental growth rates ranging from ~1 to ~10 nm/s for plagioclase in basaltic melt [62]. Brygger and Hammer [63] have observed the crystal growth of plagioclase in experimentally decompressed hydrous rhyodacite magma, reporting a maximum crystal growth rate of 0.37 nm/s. Decompressing process is not the driven force of crystallization.

The crystal growth rate observed in our test is comparable to that of the plagioclase and clinopyroxene in basaltic melt observed by Le Gall and coworkers and is larger than that reported in other studies. This larger crystal growth rate is attributed to the high concentration of Fe and Ti ions. In addition, the rapid cooling process also induced intensive driving force for crystallization (subcooling).

The crystallization process also has important implications for ISRU technologies, particularly those involving melting and solidification processes such as ISAM of lunar regolith. Partial crystallization of the lunar regolith melt is beneficial for improving the mechanical properties of the manufactured parts due to the formation of glass–ceramic microstructure. However, the degree of the crystallization should be controlled within an appropriate range. Excessive crystallization would result in the occurrence of more shrinkage pores during ISAM. These shrinkage pores in the parts can hinder the improvement of their mechanical properties and lead to the appearance of cracks during long-term service. In accordance with the crystal growth curve in Fig. 14, we can control the crystal size by adjusting the process parameters during ISAM.

### Bubbles

**Fig. 15.**
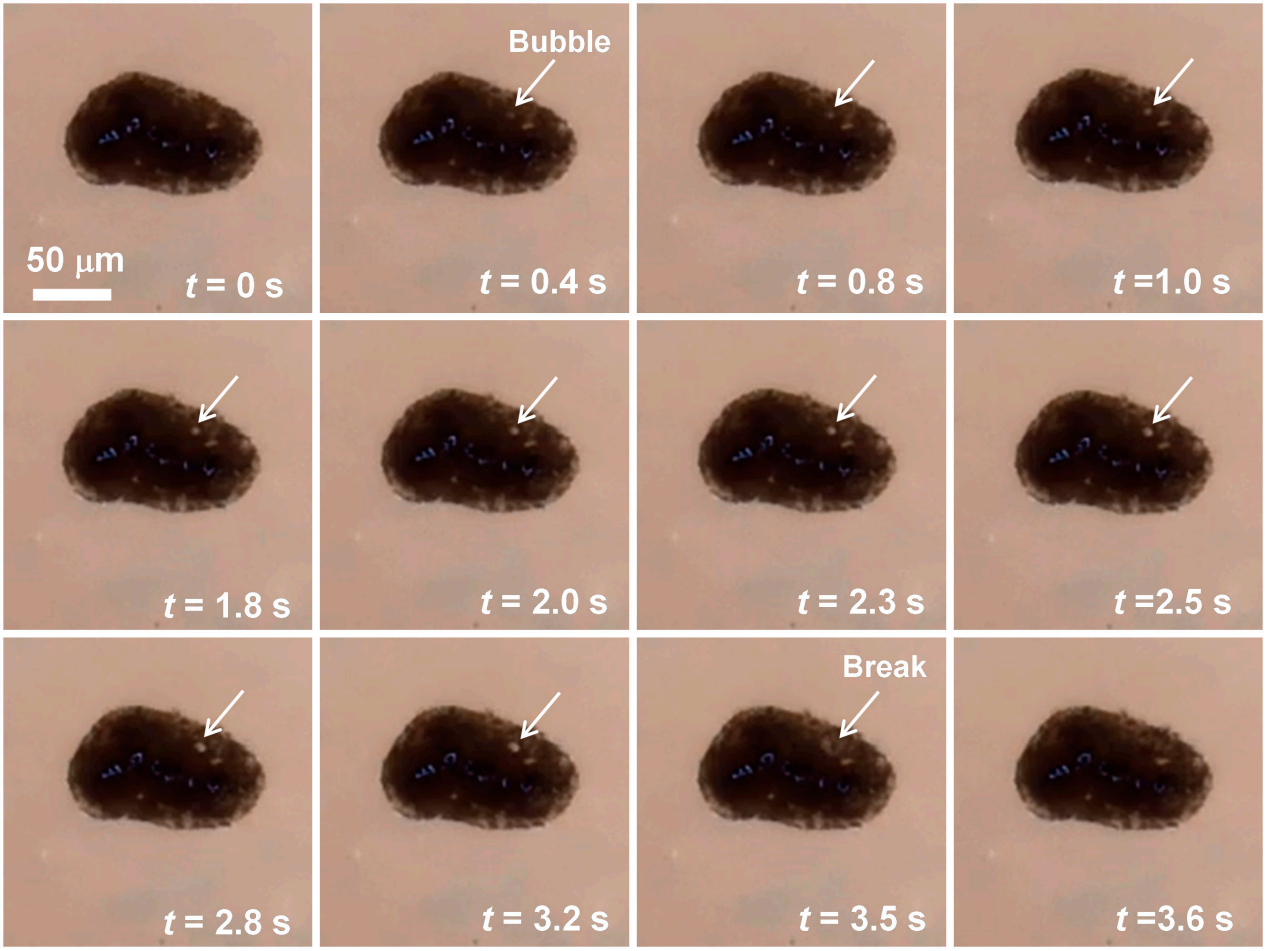
The bubble generation, growth, and release process observed in the melting of 2 agglutinate particles.

Understanding of the generation of bubbles during melting processes is critical not only for uncovering the degassing process after impacts and the formation of vesicle structures in agglutinates but also for selecting appropriate materials for in situ analysis, extracting volatiles, and controlling pore defects during ISAM. Observable bubble generation and explosion occurred during the rapid melting of agglutinates and a small amount of lunar regolith particles, indicating that agglutinates are the primary host of volatiles, rather than glass beads or ilmenite. The bubble in agglutinate melts first appeared at a temperature of approximately 1,671 K, as shown in Fig. 15. It is important to note that the particles were maintained at 1,273 K for 2 min before melting in a continuously vacuumed environment to remove any possible absorbed gas molecules. Therefore, the bubbles are caused by the volatiles encapsulated in agglutinates. The initial observable size of the bubble is roughly 1 μm. Subsequently, the bubble expanded over 3.5 s, reaching a maximum diameter of approximately 5 μm before exploding. The expansion of the bubble is attributed to the pressure difference between the bubble and the vacuum chamber. In addition, no significant pressure increase was observed when the bubble broken, implying that the gas content is low. Future melting experiments involving a larger quantity of agglutinates are necessary for gas collection and detection.

Bubbles generated by the volatiles are the primary cause of pore defects in parts obtained from ISAM. Moreover, the high-vacuum environment on the Moon would result in the severe release of volatiles. The relative long life of the bubbles observed in our experiments implied that a high energy input or a slow scanning speed during ISAM is necessary to reduce pore defects. Consequently, by controlling the temperature sequence during ISAM, we can easily adjust the size of the bubbles in 3-dimensionally printed samples to meet specific requirements, including strength, porosity, and thermal conductivity.

Our investigation into the melting and solidification processes of lunar regolith, utilizing diverse particles returned by the Chang’E-5 mission, has provided valuable insights into the complex behaviors of lunar materials under varying temperature conditions. Different lunar materials, including pyroxene, plagioclase, agglutinates, and glass fragments, exhibit unique melting and solidification behaviors. The observed crystallization patterns, featuring needle-like crystals, lath crystals, and small grains, reveal details about the composition and mineralogy of lunar materials. The formation of glass–ceramic microstructures in the solidified melts highlights the complex interplay of various lunar components. The intriguing observation of bubble generation, growth, and release during our study holds substantial inspirations for researchers exploring valuable volatiles in lunar regolith. The presence of bubbles suggests the potential existence of volatile compounds or gases that influence the lunar regolith’s behavior under varying temperature conditions. This comprehensive study sheds light on the intricate processes occurring in lunar regolith during heating and cooling, advancing our understanding of lunar geology and providing essential knowledge for future lunar exploration and resource utilization endeavors.

## Materials and Methods

### Optical, morphology, and elemental distribution

A set of optical micrographs of the lunar regolith sample CE5C0400 was acquired using a stereomicroscope (Nikon Axioscope 5). Field emission SEM (Helios G4 CX, Thermofisher) equipped with an energy-dispersive spectroscopy (EDS, X-MaxN 50, Oxford) detector was used to investigate the morphology and chemical composition of the lunar regolith sample CE5C0400. In order to protect the precious and historically significant lunar samples from contaminations or loss, individual particles were selected from the lunar regolith sample CE5C0400. Such SEM observations were carried out at a low acceleration voltage of 2 kV to suppress the accumulation of electrostatic fields during imaging, and EDS surface scans were operated at an acceleration voltage of 18 kV under high vacuum.

### Melting and solidification processes

The melting and solidification processes of the lunar regolith particles were recorded under vacuum by using a microscope (Zeiss, Axio scope 5) equipped with a high-temperature vacuum stage (Linkam, TS1500V). Individual particles were chosen from the lunar regolith sample CE5C0400 based on their different shapes and colors. The selected particles were loaded onto precleaned sapphire plates (Φ6 mm), respectively. During these loading processes, ethylene glycol was used to secure the particles onto the sapphire plates, preventing any unintended detachments. Subsequently, the sapphire plates with the loaded particles were placed in the ceramic sample cup of the stage. The stage was then sealed and vacuumized to remove the ethylene glycol. The particles underwent the following heating sequence: (a) initially heated from room temperature to 1,273 K at a rate of 80 K/min and then held at this temperature for 2 min to achieve uniformity and to remove the absorbed gas molecules; (b) subsequently heated to 1,753 K at a rate of 50 K/min, with the temperature maintained at 1,373, 1,473, 1,573, 1,673, 1,723, and 1,753 K for 4 min each; and (c) finally, cooled to room temperature at a cooling rate of 80 K/min.

## Data Availability

All data supporting the findings of this study are available within the article and its Supplementary Materials.
