# Supplementary material for: Melting and Rapid Solidification of Lunar Regolith Particles Returned by Chang’E-5 Mission
Source: Research (Wash D C). 2024 Sep 23;7:0486. doi: 10.34133/research.0486 (PMC11417502; doi:10.34133/research.0486)
Supplement: Supplementary 1 — Figs. S1 to S6 [file research.0486.f1.docx]

Supporting Information

Melting and Solidification Mechanism of Lunar Regolith Particles Returned by CE’5 Mission

Xian Zhang,^a,*^ Yiwei Liu,^a^ Shaofan Zhao,^a^ Jian Song,^a^ Wei Yao,^a,*^ Weihua Wang,^a,b^ Zhigang Zou,^a,c^ Mengfei Yang^a,d,*^

^a^ Qian Xuesen Laboratory of Space Technology, China Academy of Space Technology (CAST), Beijing 100094, China;

^b^ Institute of Physics, Chinese Academy of Sciences, Beijing 100190, China;

^c^ College of Engineering and Applied Sciences, Nanjing University, Nanjing 210093, China

^d^ China Academy of Space Technology (CAST), Beijing 100094, China;


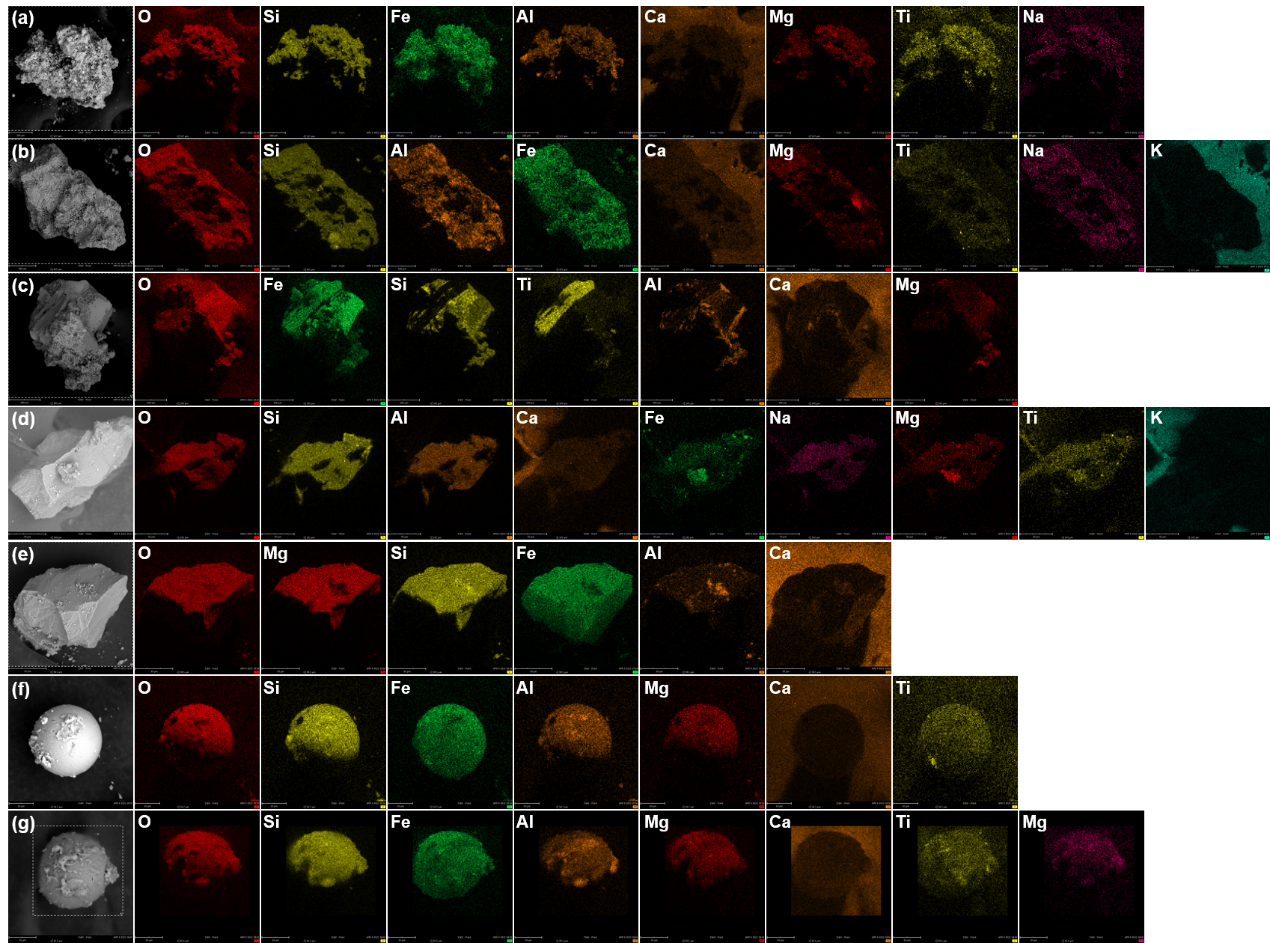


**Figure S1** SEM images and EDX mapping of (a) an aggregate particle, (b, c) black basalt-based fragments, (d) a transparent plagioclase particle, (e) a yellowish-green olivine particle, (f) a yellow glass bead, (g) a dark brown glass bead.


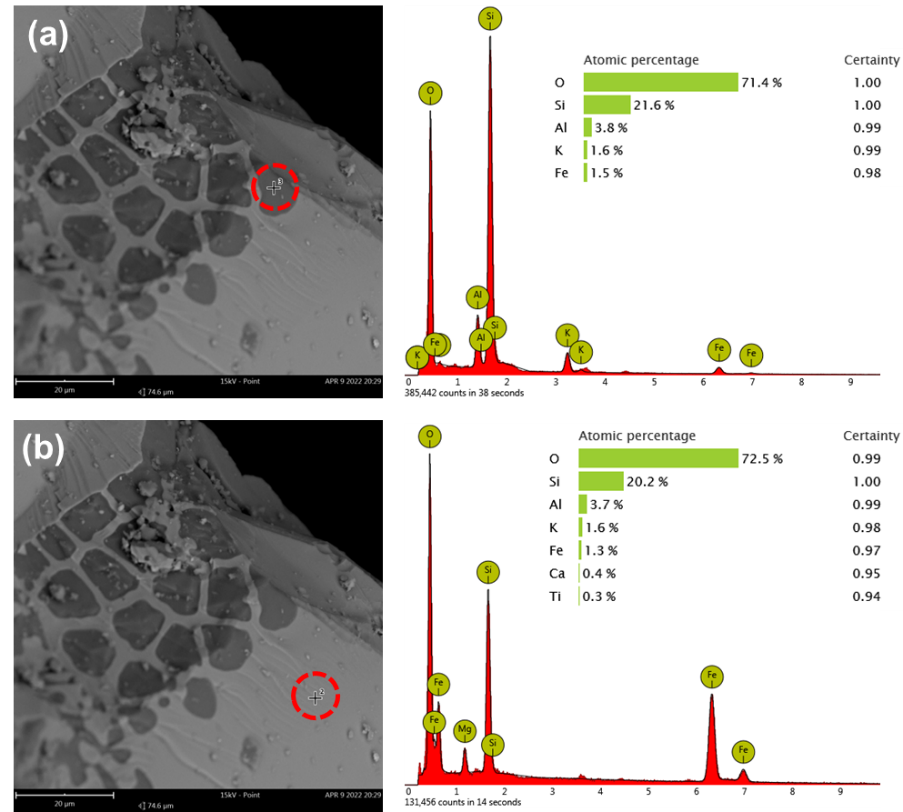


**Figure S2**. SEM and EDX spectra of the specifical region in the basalt-based fragment that composed of potassium feldspar (a) and olivine grains (b).


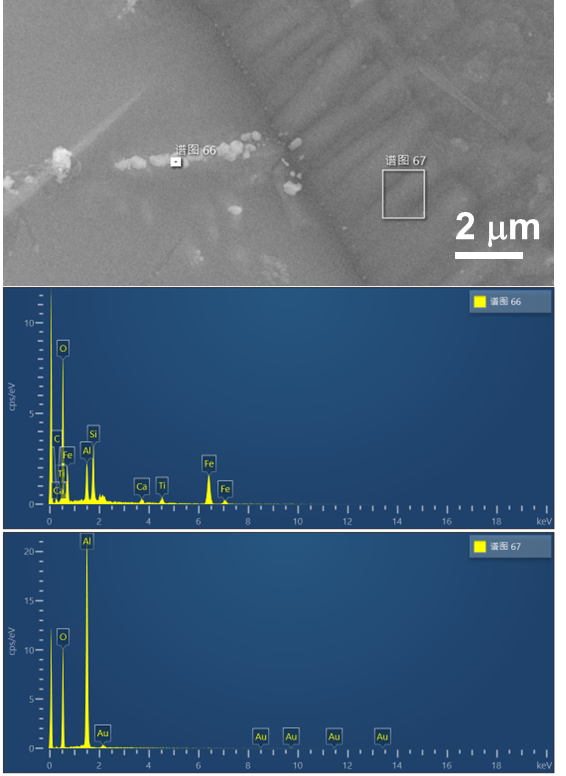


**Figure S3.** SEM image and EDX spectrum of the edge between pyroxene melt and the substrates.


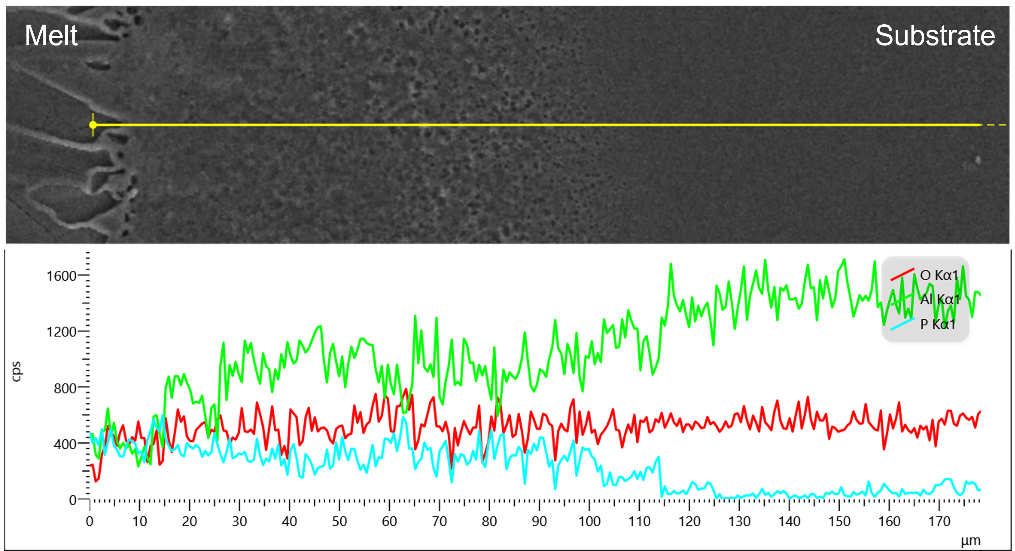


**Figure S4** Linear scanning at the edge between the melt and the substrate.


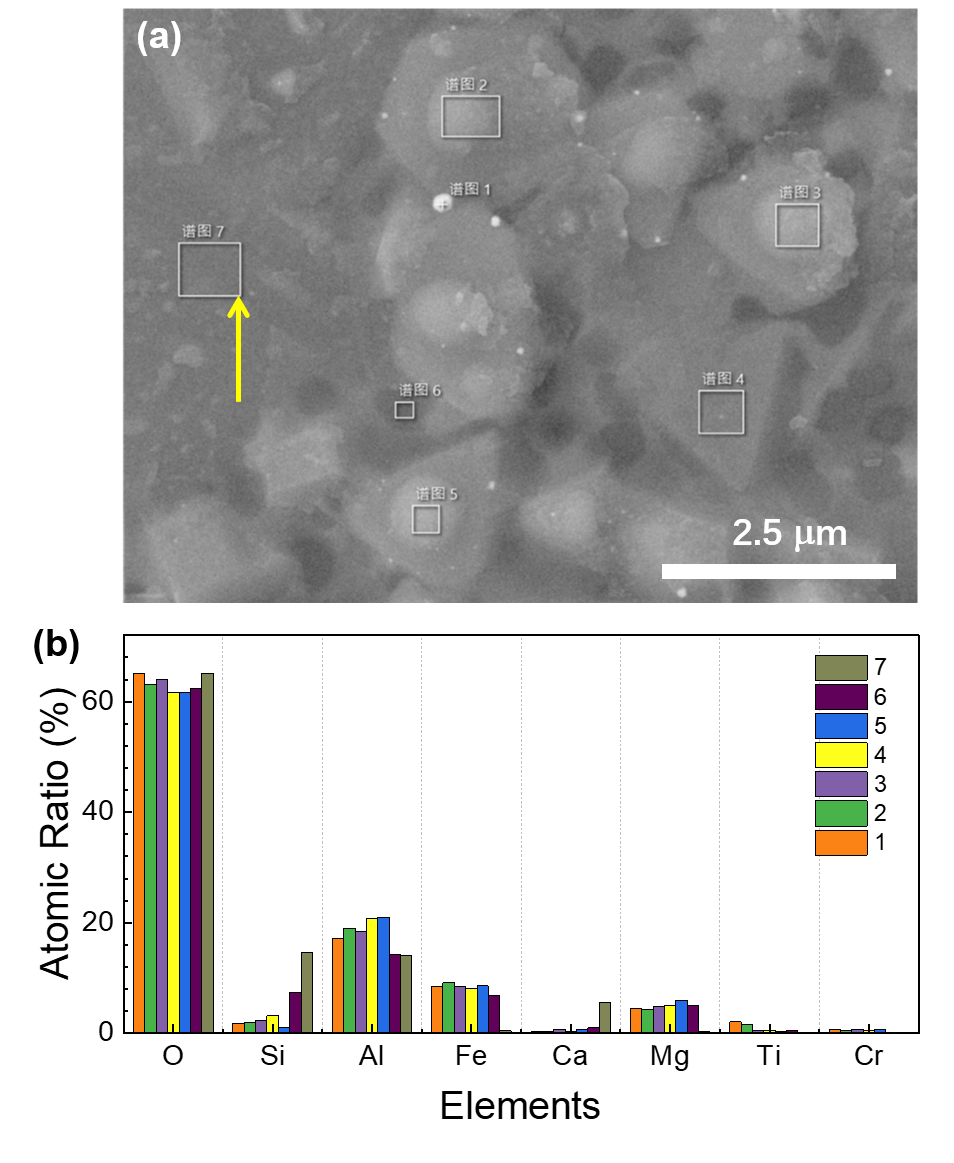


**Figure S5.** SEM (a) and elemental content (b) of the selected regions of the melt of two glass beads after cooling


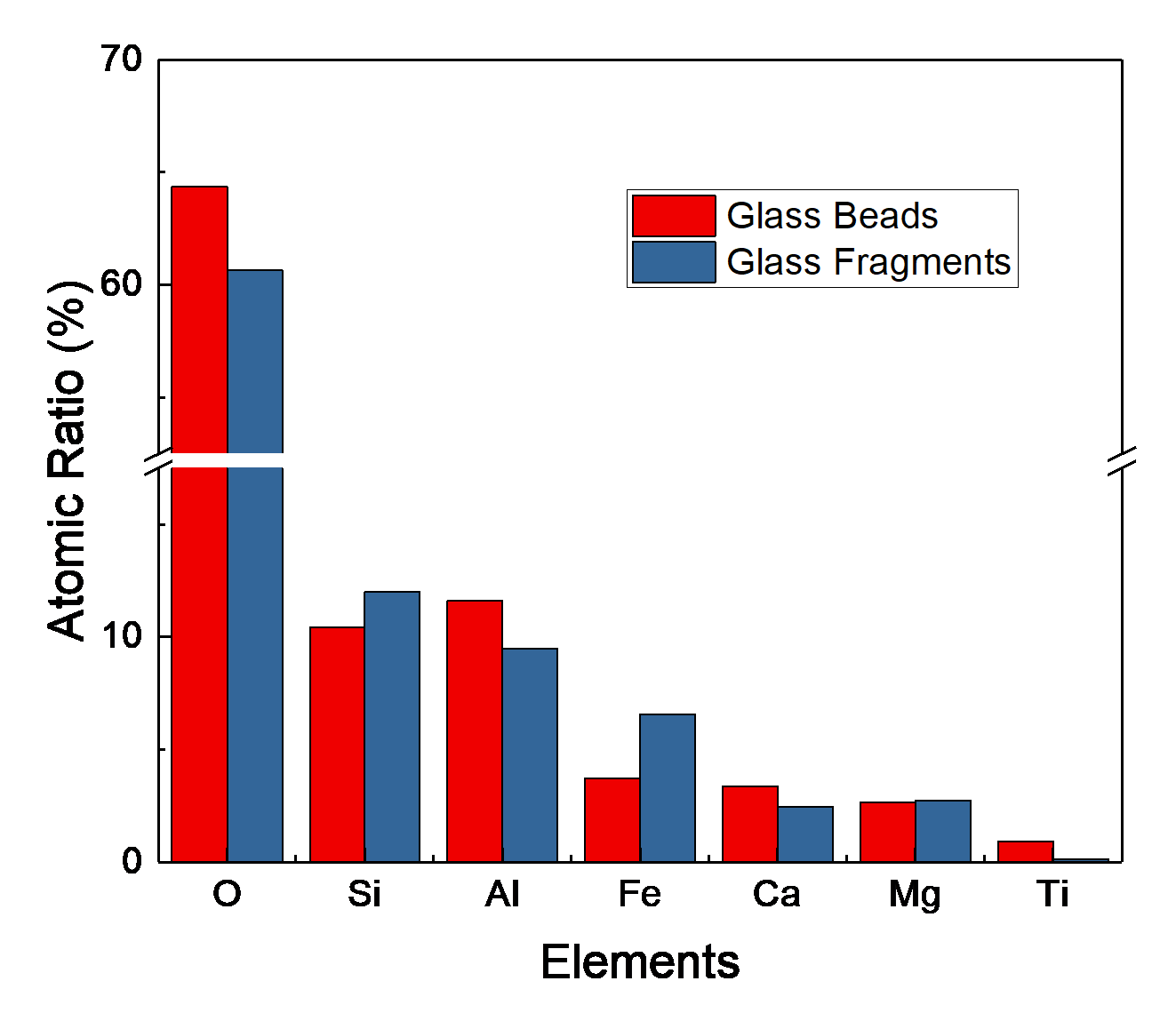


**Figure S6**. Atomic ratios of the solidified melts formed by glass beads and glass fragments.
